# Brg1 promotes liver regeneration after partial hepatectomy via regulation of cell cycle

**DOI:** 10.1038/s41598-019-38568-w

**Published:** 2019-02-20

**Authors:** Baocai Wang, Benedikt Kaufmann, Thomas Engleitner, Miao Lu, Carolin Mogler, Victor Olsavszky, Rupert Öllinger, Suyang Zhong, Cyrill Geraud, Zhangjun Cheng, Roland R. Rad, Roland M. Schmid, Helmut Friess, Norbert Hüser, Daniel Hartmann, Guido von Figura

**Affiliations:** 1 Department of Surgery, TUM School of Medicine, Klinikum rechts der Isar, Technical University of Munich, Munich, 81675 Germany; 2 Department of General Surgery, the Affiliated Zhongda Hospital, School of Medicine, Southeast University, Nanjing, 210000 China; 3 Institute of Molecular Oncology and Functional Genomics, Department of Medicine II and TranslaTUM Cancer Center, Klinikum rechts der Isar, Technical University of Munich, Munich, 81675 Germany; 4 Institute of Pathology, TUM School of Medicine, Klinikum rechts der Isar, Technical University of Munich, Munich, 81675 Germany; 5 Department of Dermatology, Venereology, and Allergology, University Medical Center and Medical Faculty Mannheim, Heidelberg University and Center of Excellence in Dermatology, Mannheim, 68135 Germany; 6 Department of Medicine II, TUM School of Medicine, Klinikum rechts der Isar, Technical University of Munich, Munich, 81675 Germany

## Abstract

Brahma-related gene 1 (Brg1), a catalytic subunit of the SWItch/Sucrose Non-Fermentable (SWI/SNF) complex, is known to be involved in proliferative cell processes. Liver regeneration is initiated spontaneously after injury and leads to a strong proliferative response. In this study, a hepatocyte-specific Brg1 gene knockout mouse model was used to analyse the role of Brg1 in liver regeneration by performing a 70% partial hepatectomy (PH). After PH, Brg1 was significantly upregulated in wildtype mice. Mice with hepatocyte-specific Brg1 gene knockout showed a significantly lower liver to body weight ratio 48 h post-PH concomitant with a lower hepatocellular proliferation rate compared to wildtype mice. RNA sequencing demonstrated that Brg1 controlled hepatocyte proliferation through the regulation of the p53 pathway and several cell cycle genes. The data of this study reveal a crucial role of Brg1 for liver regeneration by promoting hepatocellular proliferation through modulation of cell cycle genes and, thus, identify Brg1 as potential target for therapeutic approaches.

## Introduction

The liver has a unique regenerative capacity to regain its size, architecture, and function in response to the loss of mass caused by a variety of injuries^1^. This regenerative capacity provides the basis for a potentially satisfying clinical outcome for patients after a serious hepatic injury, cancer resection, or living donor liver transplantation. The regenerative capacity is often reduced when concomitant liver disease, such as liver fibrosis or non-alcoholic fatty liver disease (NAFLD), is present. To promote liver regeneration therapeutically, it is therefore important to decipher the molecular mediators that regulate liver regeneration.

Liver regeneration starts with a well-organised and complex series of signals, which are generated by cytokines and growth factors^2^. The use of the rodent partial hepatectomy (PH) model described originally by Higgins and Anderson^3^ resulted in a better understanding of the three sequential and critical steps leading to liver regeneration^4^. Firstly upon PH, hepatocytes exit their quiescent and highly differentiated state in order to rapidly re-enter the cell cycle (priming phase). Secondly, with the help of mitogens, hepatocytes enter the cell cycle and progress beyond the restriction point to G1 phase and M-phase in order to proliferate and compensate for the removed mass (proliferation phase)^5^. After approximately two cell cycles of hepatocyte replication, cells terminate proliferation under the control of negative factors (termination phase)^6^. Finally, liver mass is restored to the size before hepatectomy, and liver morphology is gradually rearranged^7^.

Epigenetic mechanisms are a relevant regulatory component of many biological processes, including organ regeneration^8^. A crucial epigenetic regulator is the SWItch/Sucrose Non-Fermentable (SWI/SNF) complex, a large multi-subunit chromatin remodelling complex^9^, that consists of approximately 15 subunits^10^. The mammalian SWI/SNF complex family is further subdivided into two major complexes, the brahma related gene 1 (Brg1)-associated factor complex (BAF) and the polybromo Brg1-associated factor (PBAF) complex^11^. While the catalytic subunit Brahma (Brm) is used only for BAF complexes, Brahma related gene 1 (Brg1) is a subunit of both mammalian SWI/SNF complexes^12^. Recently, an important role for this complex could be shown for liver regeneration. It was revealed that the subunit Arid1a plays a prominent role in the context of liver regeneration by impairing liver regeneration, mainly due to a positive modulation of target gene transcription that repress proliferation^13^. However, the exact function of the SWI/SNF complex and, in particular, its catalytic ATPase subunits in liver regeneration remain unclear.

The main catalytic ATPase subunit of the SWI/SNF complex Brg1 is essential for embryogenesis and organogenesis as well as gene expression and development of different tissues^14–20^. Besides its role as an epigenetic regulator Brg1 is also known to directly bind to the promoter of different target genes to activate/silence gene expression. Hereby, Brg1 functions as a transcription factor itself for various genes^21^. The exact role of Brg1 in the context of the regulation of gene expression is so far not completely understood. Furthermore, in numerous malignant tumors, Brg1 is mutated and overexpressed^22^. A previous study from our group demonstrated that Brg1 is overexpressed in hepatocellular carcinoma (HCC) and positively promotes proliferation^23^. In doing so, Brg1 regulates different cell cycle genes, mainly the genes of the cyclin family. Regeneration studies of other organs revealed that the repression of cyclin-dependent kinase (Cdk) inhibitors by Brg1 is the driving force for regeneration^24,25^. Furthermore, the interaction between Brg1 and Brm in different phases of liver injury and regeneration contributes essentially to liver regeneration^26^. Whereas Brm dominates during the late injury phase and initiation of regeneration phase, Brg1 is the main catalytic subunit of the SWI/SNF complex during the injury and regeneration phase^26^. However, the precise role of Brg1 on proliferation during liver regeneration after liver injury as well as the signaling pathway remain unclear.

The aim of this study was to investigate the role of Brg1 in hepatocytes during liver regeneration in mice and to analyse molecular signaling pathways modulated by Brg1. By using a mouse model with hepatocyte-specific knockdown of Brg1, this study reveals an important function of Brg1 during liver regeneration by promoting hepatocellular proliferation through modulation of cell cycle genes.

## Results

### Brg1 expression increases after PH

**Figure 1:**
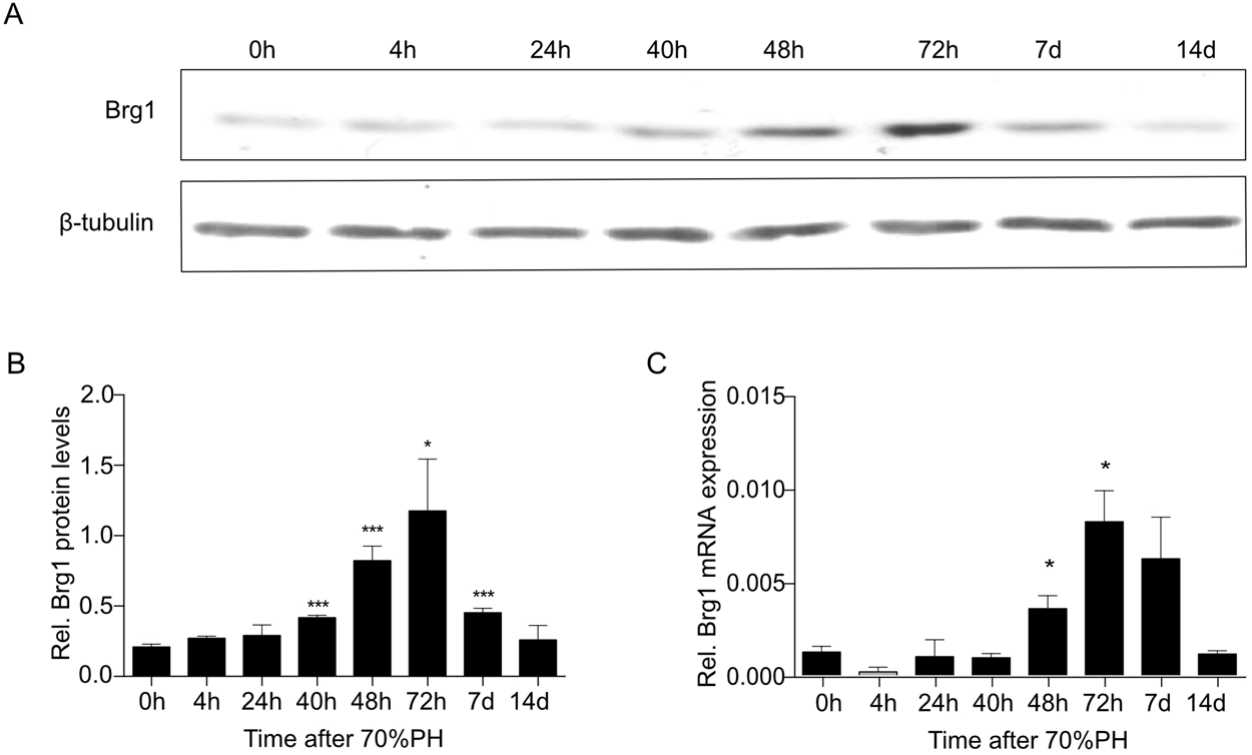
Brg1 expression during liver regeneration progression after PH in mice. (**A,B**) Protein expression of Brg1 in the liver after PH analysed by Western blot. Representative gels (**A**) and densitometric analyses (**B**) are depicted, n = 3. (**C**) mRNA expression of Brg1 after PH, n = 3.

Hepatic Brg1 expression was assessed, before and at different time points after PH in mice to explore the regulation of Brg1 during liver regeneration. Weak Brg1 expression was observed in the healthy liver before PH. At the early stage of liver regeneration (within 24 h after PH), there was no significant alteration of the Brg1 expression on mRNA or protein levels (Fig. 1A–C). Both the protein and mRNA levels of Brg1 started to increase gradually when liver regeneration proceeded. Three days after PH, protein and mRNA levels of Brg1 were highest (Fig. 1A–C). At day 2 after PH, Brg1 protein expression was increased by approximately 4.0-fold; at day 3 after PH, Brg1 protein expression was increased by approximately 6.3-fold compared to the protein level before PH. However, after peak expression on day 3 after PH, Brg1 expression on day 7 declined to equalize the expression level seen before PH.

### Brg1 knockout mice show normal liver development

Hepatocyte-specific Brg1 knockout mice were generated by breeding Brg1-floxed mice with albumin-Cre transgenic (AlbCre) mice, which express Cre recombinase specifically in hepatocytes under the control of the albumin promoter^27^. This finally generated AlbCre-Brg1^fl/fl^ mice (hereafter termed Brg1 KO mice). Brg1^fl/fl^, Brg1^fl/−^ mice obtained from the same breeding were used as controls (hereafter termed Control mice).

**Figure 2:**
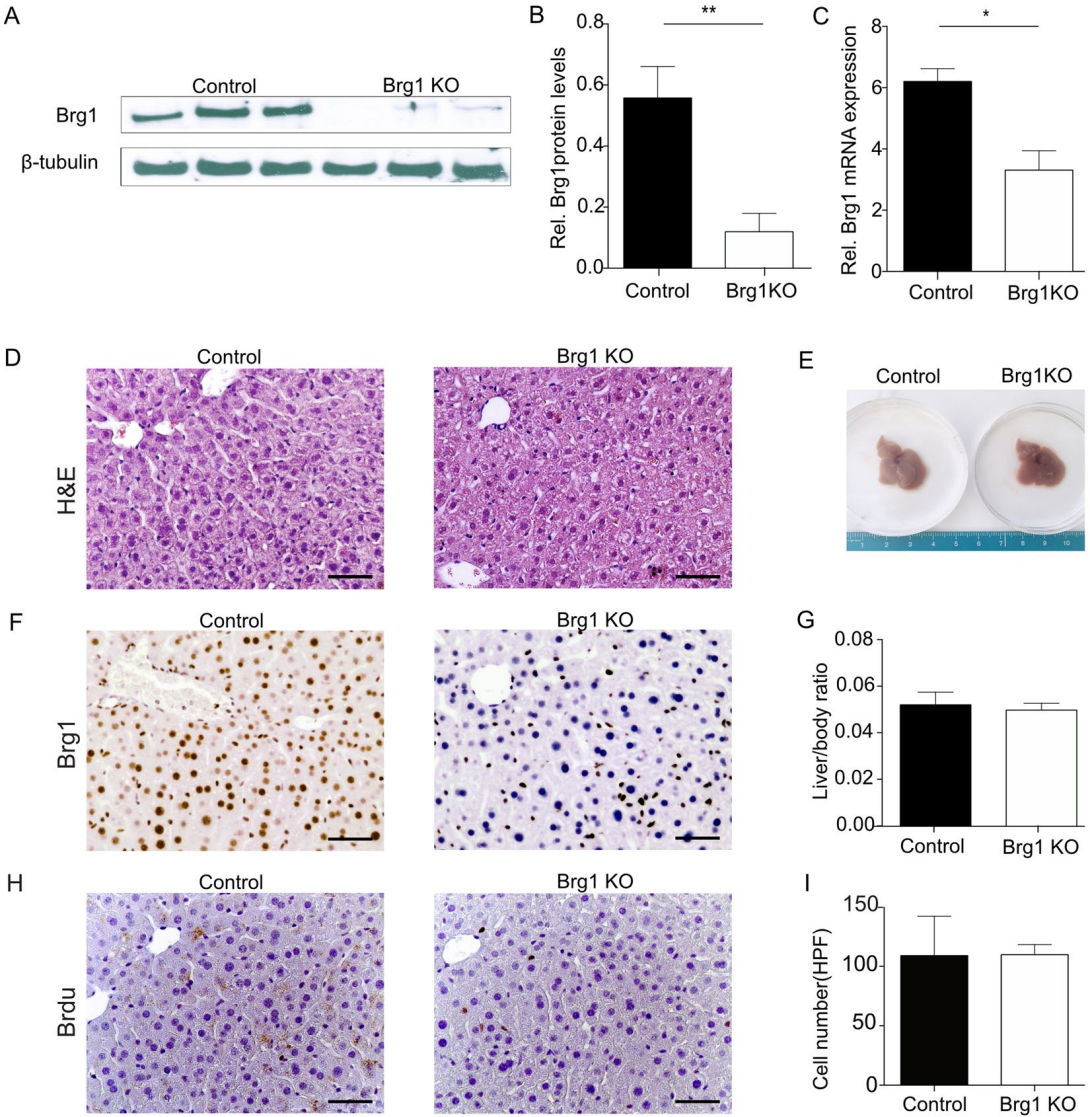
Brg1 knockout mice show normal liver development. (**A,B**) Protein expression of Brg1 in the liver of 2-month-old Control and Brg1 KO mice analysed by Western blot. Representative gels (**A**) and densitometric analyses (**B**) are depicted. n = 3. (**C**) mRNA expression of Brg1 of 2-month-old Control and Brg1 KO mice in the liver analysed by qPCR, n = 3. (**D**) H&E staining of the liver of 2-month-old Control and Brg1 KO mice. (**E**) Representative liver morphology of 2-month-old Control and Brg1 KO mice. (**F**) Immunohistochemistry for Brg1 of the liver of 2-month-old Control and Brg1 KO mice. (**G**) Liver to body weight ratios of 2-month-old Control and Brg1 KO mice. n = 6. (**H**) Representative immunohistochemistry images for BrdU of 2-month-old Control and Brg1 KO mice livers. (**I**) Quantification of cell number (HPF) of 2-month-old Control and Brg1 KO mice, n = 6.

For the following analysis, the livers of 2-month-old Brg1 KO mice and Control mice were compared. In the liver tissue, Brg1 KO mice showed a significant decrease of about 80% of the protein and RNA expression of Brg1 compared to Control mice (Fig. 2A–C). As the albumin promoter is active in hepatocytes but not in other liver cells, there was still expression of Brg1 in the non-hepatocellular liver tissue of Brg1 KO mice (Fig. 2F). Brg1 KO mice were healthy and possessed normal transaminase levels (Fig. S1A). Liver structure and metabolic zonation were similar in both the Brg1 KO and Control groups (Fig. 2D,E, Fig. S1D–F). The body weight, the liver weight, and the liver to body weight ratio of Brg1 KO mice were not significantly different from those of Control mice (Fig. 2G, Fig. S1B,C). The proliferation rate and hepatocyte cell size in the liver tissue of 2-month-old Brg1 KO and Control mice were compared by BrdU staining and microscopy (Fig. 2H,I). Neither the proliferation ratio nor the hepatocyte cell size were significantly different in Brg1 KO mice compared to Control mice (Fig. 2H,I).

### Brg1 loss impairs liver regeneration after PH

**Figure 3:**
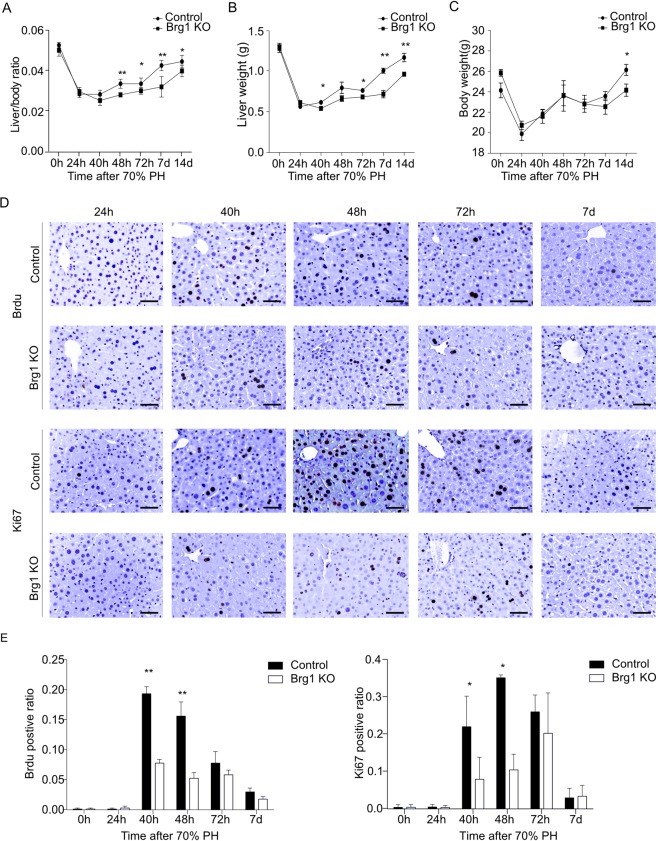
Lack of Brg1 delays recovery of liver tissue after PH. (**A–C**) Liver to body weight ratios (**A**), liver weight (**B**) and body weight (**C**) were determined at the indicated time points after PH, n = 6. (**D**) Representative immunohistochemical images for BrdU and Ki67 at the indicated time points. (**E**) Quantification of positive staining of BrdU and Ki67 in hepatocyte nuclei at the indicated time points, n = 6.

To investigate the role of Brg1 in liver regeneration, a surgical resecting of two-thirds of the liver was performed. After liver resection, the liver tissues of both Brg1 KO and Control mice were vital and exhibited neither necrosis nor inflammation as analysed by H&E staining (Fig. S2A). Liver regeneration was markedly impaired in Brg1 KO mice, showing a significantly reduced liver to body weight ratio and liver weight at 48 h, 72 h, 168 h, and 336 h after PH compared to Control mice **(**Fig. 3A–C**)**. Concerning the liver to body weight ratio and liver weight, liver regeneration of Control mice was nearly completely terminated 168 h after PH, whereas Brg1 KO mice showed a prolonged time for recovery of the same liver mass that even after 336 h did not reach the levels of Control mice at 168 h (Fig. 3A–C). ALT levels strongly increased after PH in both Brg1 KO and Control mice, but no significant difference of ALT expression was seen between both genotypes (Fig. S2B).

**Figure 4:**
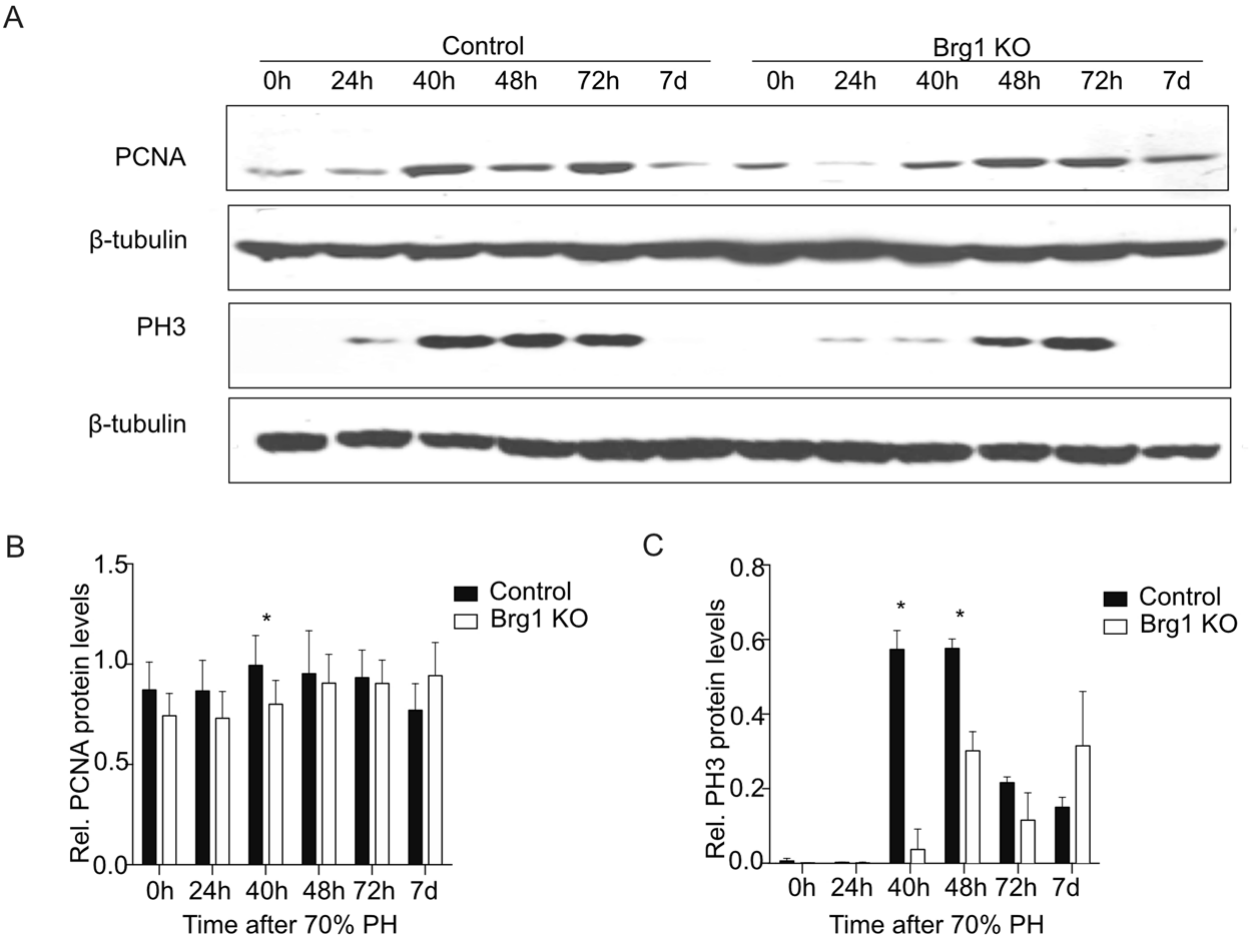
Lack of Brg1 impairs liver proliferation after PH. (**A–C**) Analysis of protein expression of PCNA and PH3 in the liver by Western blot. Representative gels (**A**) and densitometric analyses (**B,C**) are depicted n > 3.

Extensive proliferation of liver parenchymal cells is required to restore liver tissue^28^. In order to analyse proliferation, staining of Ki67 as a marker for the mid-G1 phase to the end of mitosis and incorporation of 5-bromo-2′-deoxyuridine (BrdU) for the S phase were performed. Analysing both BrdU and Ki67 immunoreactivity, active proliferation was observed in hepatocytes in Control mice from 40 h to 48 h post-PH, while proliferation was significantly decreased in Brg1 KO mice at these time points (Fig. 3D,E). The cell cycle-dependent proliferating cell nuclear antigen (PCNA) expression is commonly used as an accurate and reproducible marker of liver regeneration, whereas phosphorylated histone H3 (pH3) is a G2/M marker. Therefore, the protein expression of PCNA and pH3 were evaluated by Western Blot (WB). In line with the results from the Ki67 and Brdu staining, it was shown that in the Brg1 KO group the expression of PCNA and pH3 was significantly lower than in the Control group at 40 h after PH for both PCNA and pH3 and at 48 h after PH for pH3 (Fig. 4A–C). Taken together, these results demonstrate that Brg1 is required for hepatocyte proliferation during recovery of liver tissue after PH.

### Loss of Brg1 impairs liver regeneration by modulating the cell cycle pathway

**Figure 5:**
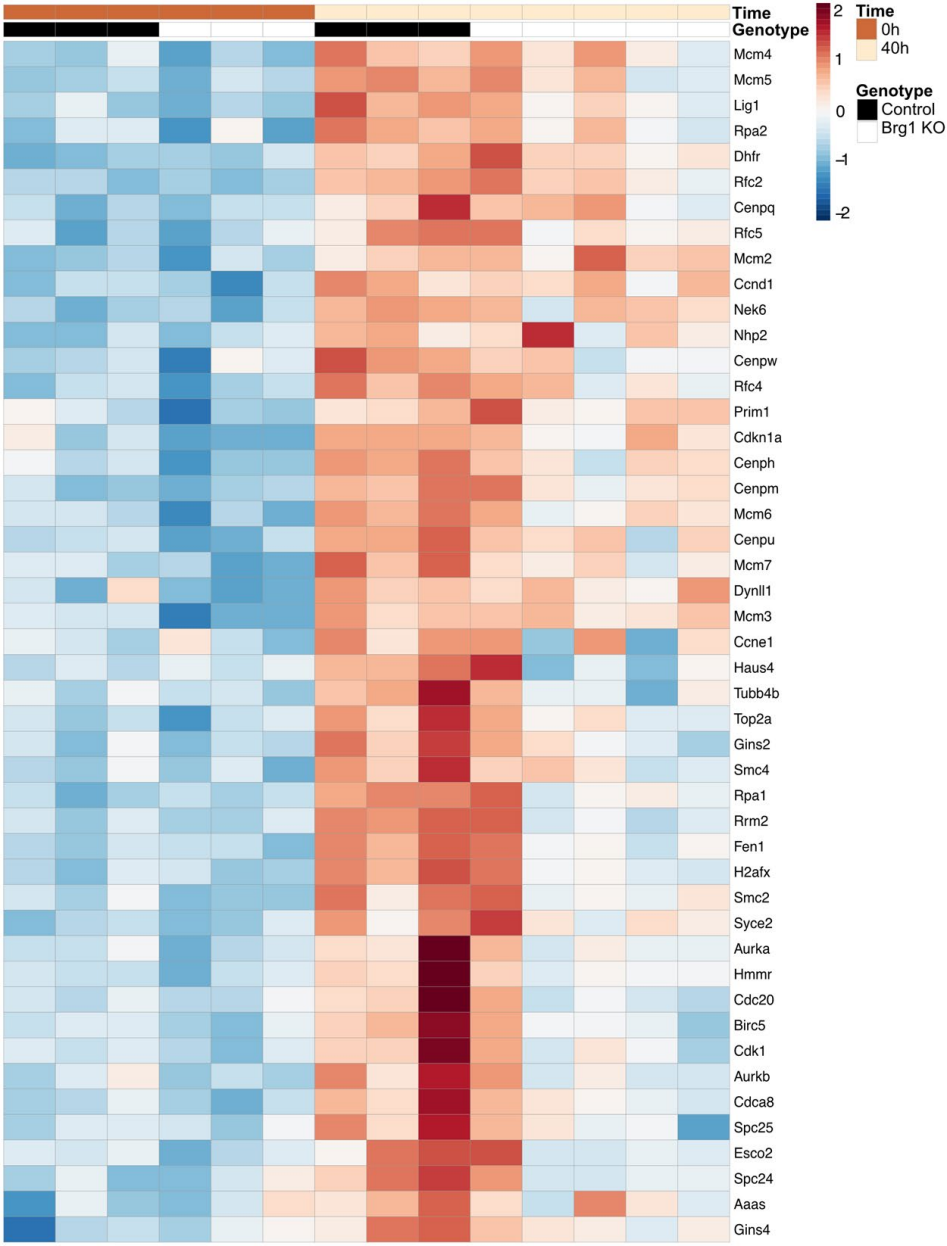
Loss of Brg1 impairs liver regeneration by modulating the cell cycle pathway. Heatmap of significantly regulated cell cycle related genes. Shown is the z-scaled gene expression for genes, being significantly regulated between the time points 0 h and 40 h, within both the Control and Brg1 KO group, and overlap with the cell cycle pathway annotation from Reactome.

**Figure 6:**
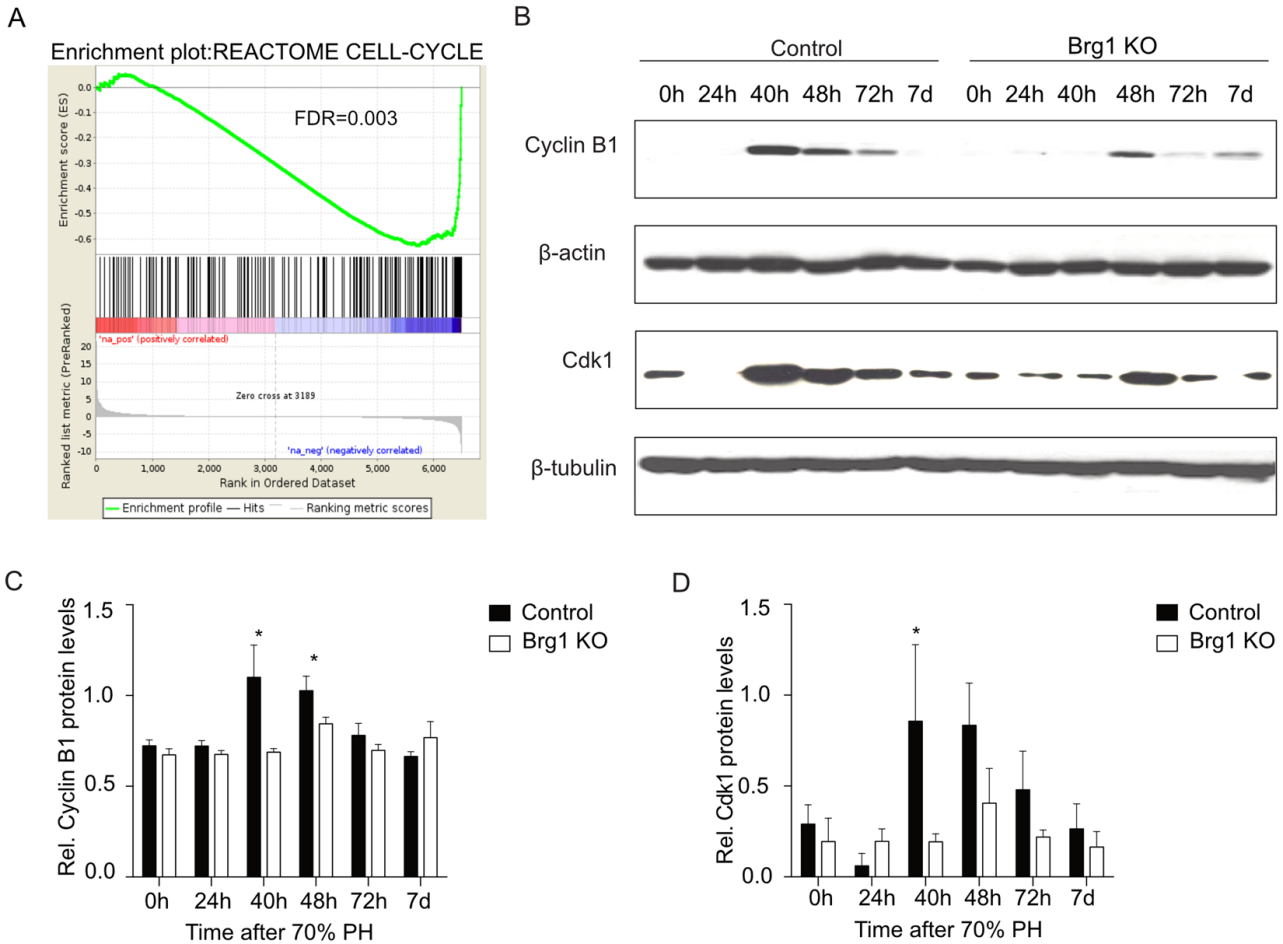
Impaired cell cycle progression in mice with defective Brg1. (**A**) GSEA enrichment plot for the cell cycle pathway. GSEA analysis was conducted for detecting differences between the Brg1 KO and the Control group at 48 h post PH. The cell cycle pathway is significantly associated with the Brg1 KO genotype (FDR = 0.0003). (**B–D**) Protein expression of Cyclin B1 and Cdk1 in the liver after PH was analysed by Western blot. Representative gels (**B**) and densitometric analyses (**C,D**) are depicted, n > 3.

**Figure 7:**
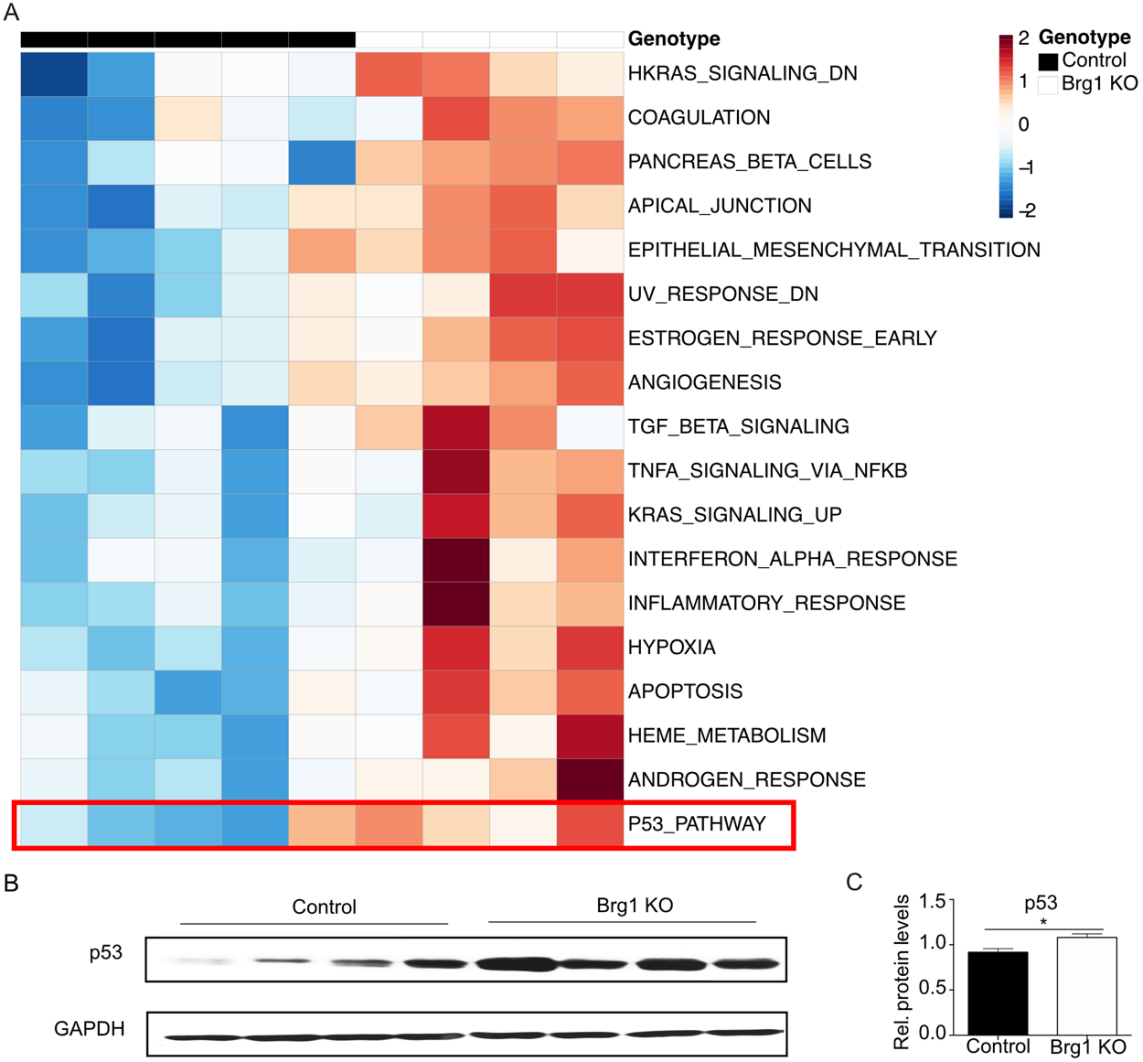
Brg1 regulates cell cycle via p53 pathway. (**A**) Heatmap of GSVA analysis results at 48 h after PH. The z-transformed enrichment scores for significantly associated pathways with the Brg1 genotype are shown. (**B,C**) Analysis of protein expression of p53 by Western blot in the liver at 48 h post PH. Representative gels (**B**) and densitometric analyses (**C**) are depicted n = 4.

To identify genes regulated by Brg1 during liver regeneration, RNA-sequencing (RNA-seq) on liver extracts was performed. Four time points (4 h, 24 h, 40 h and 48 h) post-PH were screened and these expression levels were compared to the baseline expression pre-PH (Table S1). At time points 40 h and 48 h, both Brg1 KO group and Control group showed an upregulation of genes related to the cell cycle pathway, such as Ccnb1, Ccnb2, Cdk1 and Cdc20 (Table S2). Interestingly, this regulation is much less pronounced in the Brg1 KO group compared to the Control group (Figs 5, S3). In accordance with this observation, a Gene Set Enrichment Analysis (GSEA) analysis for differences between genotypes at time point 48 h also shows a significant downregulation of the cell cycle pathway in the Brg1 KO group (Fig. 6A). Based on the RNA-seq results, it was demonstrated that Brg1 impairs liver regeneration by modulating the cell cycle pathway. In particular, Cyclin B and Cdk1 showed a significantly positive modulation by Brg1 after PH. Therefore, the protein levels of both Cyclin B and Cdk1 during the liver regeneration phase were analysed by Western blot showing a significant lower expression of Cyclin B1 and Cdk1 in the Brg1 KO group compared to the Control group 40 h and 48 h after PH (Fig. 6B–D). To further investigate possible mechanisms of the cell cycle activation, we performed Gene Set Variation Analysis (GSVA) on each sample at 48 hr post PH. In contrast to other Gene Set Testing methods it can account for heterogenous pathway activity, e.g. pathways are activated by different genes in different samples within an experimental group. It was shown that in particular the p53 pathway is upregulated in the Brg1 KO group compared to the Control group (Fig. 7A). P53 is an important factor that positively modulates the cell cycle by different mechanisms. The expression of p53 on protein level was analysed and a significant upregulation of p53 in Brg1 KO group compared to Control group was demonstrated (Fig. 7B,C). This data confirmed the finding of GSVA of RNA-sequencing. Taken together, these results indicate that in hepatocytes Brg1 influences the cell cycle by modulating cyclins, in particular Cyclin B1 and Cdk1.

## Discussion

Liver regeneration after PH requires extensive and coordinated proliferation of hepatocytes. Recent studies have indicated that mutations causing Brg1 dysfunction are related to various diseases that are characterised by aberrant cell proliferation^14,15^. However, the underlying mechanisms by which Brg1 regulates cell proliferation are still not fully understood. In this study, the essential role of Brg1 in liver regeneration was investigated and it was demonstrated that Brg1 is required for the proliferative response of hepatocytes during this process.

In this study it was shown that Brg1 mRNA and protein levels were transiently upregulated during liver regeneration and Brg1 KO mice had a significantly lower proliferation rate and liver to body ratio than the Control group 48 h after PH. Therefore, these findings underline the important role of Brg1 for tissue regeneration and proliferation in the liver and they are in line with previous studies that showed a crucial role of Brg1 for tissue regeneration and proliferation in other organs^24,25^. The data of this study are also in accordance with the expression studies of Sinha *et al*. that suggested an important role of Brg1 during the injury and regeneration phase of the liver after thioacetamide-induced liver injury. A specific role of Brg1 on proliferation was also shown in HCC by our group^23^.

The RNA-seq results of this study showed that in the proliferation phase of liver regeneration, the cell cycle pathway is impaired in Brg1 KO mice. Cyclins, especially Cyclin B1, which is induced during the G2 phase of the cell cycle, was decreased in Brg1 KO mice. Consistent with these findings, the expression of the Cdk1 protein that forms a complex with Cyclin B and plays a key role for advancing the cell cycle to the M phase, was also significantly reduced. Similarly, regulation of cell cycle genes by Brg1 was also identified as the main force to promote tissue regeneration and proliferation in other organs^24,25^. However, in these studies an inverse interaction of different cyclin-dependent kinase inhibitors and Brg1 in order to regulate the cell cycle was identified. Thus, Brg1 regulates the cell cycle in a cell- and context-dependent fashion.

The importance of Cyclin B during liver regeneration was also recently demonstrated^13^. In contrast to our study, the suppression of the SWI/SNF subunit Arid1a leads to an increase of Cyclin B and promotes liver regeneration^13^. In addition, complete ablation of Arid1a improves the regenerative capacity after liver injury, mainly due to a suppression of target genes that inhibit proliferation^13^. This contrary role of individual subunits of the SWI/SNF complex in liver regeneration remains unclear. A possible explanation may be the pattern of genes that each subunit binds. Whereas Brg1 may mainly interact positively with genes promoting liver regeneration, Arid1a may regulate predominantly genes repressing proliferation and regeneration^13^.

In this study the regulation of p53 pathway by Brg1 in liver regeneration was identified. These findings are in line with previous studies that showed an inverse correlation of Brg1 and p53 in order to regulate proliferation and cell cycle arrest^29–32^. Hereby, Brg1 negatively regulates p53, which is an important gene that modulates cell cycle by different mechanisms on protein level^32^. Interestingly, it is shown that p53 can also bind directly to the Cyclin B1 promoter and therefore inhibit Cyclin B1 transcription^33^. Consequently, during liver regeneration Brg1 KO mice used in this study show an upregulation of p53 protein caused by a lack of Brg1 level. Increased p53 protein expression can lead to a decrease of Cyclin B and subsequently reduced cell proliferation. In addition, not only an indirect regulation of cyclins by Brg1 via p53 is reported. A direct binding of Brg1 to the promoter of Cyclin B was also shown^34^. Through this, Brg1 is able to regulate cell cycle by direct regulation of the expression of Cyclin B independent of upstream pathways. Taken together, in our study we were able to show that Brg1 regulates cell cycle by modulating expression of cyclin family, presumably in a direct and p53-dependent indirect manner. Interestingly, in a study by Li and colleagues liver proliferation was mainly modulated by Brg1 via activation of β-catenin activity indicating an additional Brg1-dependent pathway that may impact liver regeneration^35^.

In summary, this study demonstrates the critical role for Brg1 in the regulation of liver regeneration and proliferation by modulating cell cycle genes. The findings of this study highlight the specific role of Brg1 for liver regeneration after liver injury and demonstrate a positive modulation of cyclins and cyclin-dependent kinases by Brg1. However, the exact mechanisms that regulate the expression of Brg1 and the interaction of Brg1 with target gene sites during liver regeneration are still not fully understood and require further investigation. Therefore, Brg1 represents a novel potential therapeutic target to promote liver proliferation after tissue damage.

## Methods

### Animals

All mice were housed in specified pathogen-free facilities (ZPF, Klinikum rechts der Isar, Munich, Germany). Mice with a homozygous deficiency of Brg1 were generated by intercross of Brg1^fl/fl^ and AlbCre single mutant mice on a mixed genetic background. Corresponding controls (Brg1^fl/fl^, Brg1^fl/−^) were provided. All experiments were performed in an age- and sex-controlled fashion unless otherwise noted in the figure legends.

### Partial hepatectomy

Male mice at the age of 8 to 10 weeks were subjected to two-thirds partial hepatectomy using standard procedures according to published protocol^36^. Ligation and resection of the middle and left lobes were performed under isoflurane inhalation anesthesia. Partial hepatectomy was performed between 8 and 10 am. At the indicated time points, mice were sacrificed, blood was collected by cardiac puncture, and remnant livers were fixed in 4% formaldehyde or snap frozen in liquid nitrogen immediately after explantation and stored at −80 °C until further use. Animal experiments were institutionally approved by the District Government of Upper Bavaria (AZ 55.2.1.54-2532-125-2015) and performed in accordance with the relevant guidelines and institutional regulations.

### Western blotting

Liver samples were lysed in RIPA buffer (Cell Signaling Technology) supplemented with protease and phosphatase inhibitors. Protein concentration was determined by using the PierceTM BCA Protein Assay Kit (Thermo Scientific). Lysates were separated by SDS-PAGE and transferred to a Whatman Protran BA85 membrane (GE Healthcare). Membranes were incubated with the following primary antibodies overnight: Brg1 (Santa Cruz Biotechnology), β-tubulin (Abcam), β-actin(Santa Cruz Biotechnology), PCNA (Cell Signaling Technology, Inc.), pH3 (Cell Signaling Technology, Inc.), Cyclin B1 (Cell Signaling Technology, Inc.), Cdk1 (Cell Signaling Technology, Inc.), GAPDH (Santa Cruz Biotechnology), p53 (Leica Biosystems), then incubated with Secondary antibody goat-anti-rabbit-HRP or goat-anti-mouse-HRP (Promega) for 1 h. Antibody binding was visualized using the Pierce™ ECL western blotting detection system (GE Healthcare). Densitometric analysis was performed using the ImageJ software (https://imagej.nih.gov/ij/).

### Quantitative reverse transcriptase PCR

RNA was prepared using the RNeasy Mini Kit (Qiagen N.V.). First-strand cDNA was synthesised from 1 mg total RNA using the QuantiTect Reverse Transcription Kit (Qiagen N.V.). Quantitative RT-PCR (Reverse transcription–polymerase chain reaction) analyses were performed using the Universal Probe Library (Roche Diagnostics). RNA levels were normalized to those of GADPH and are depicted as fold difference relative to liver samples of untreated mice. Accumulation of PCR amplicons was quantified on a LightCycler 480 Real-Time PCR system (Roche Diagnostics).

### Histology and Immunohistochemistry

Mice underwent intraperitoneal injections of 100 µg/g BrdU (Roche Applied Science) 2 h before sacrifice. Liver samples were fixed overnight in 4% paraformaldehyde, dehydrated in a graded alcohol series, and embedded in paraffin. H&E and immunohistochemistry stainings were performed on 3 μm archived liver sections. Primary antibodies used: Brg1 (Santa Cruz Biotechnolog), Ki-67 (Abcam) and BrdU (BD). Sections were stained using diaminobenzidin (DAB, Liquid DAB+ Substrate Chromogen System, DAKO). For each animal, five random high power fields were counted and the fraction of stained hepatocyte nuclei was calculated.

### Immunofluorescence

Deparaffinization and rehydration of paraffin sections (1 µm) was performed according to standard protocols. Antigen retrieval was carried out with epitope retrieval solution (Zytomed Systems, Germany) at pH 6. The first antibody was incubated over night at 4 °C, the secondary antibody was applied for 1 h at room temperature after three washing steps with PBS. Sections were mounted with Dako fluorescent mounting medium (Dako, Agilent technologies, USA) and photographed with ECLIPSE Ni-E microscope (Nikon). Antibodies: goat anti-Lyve1 (AF2125, R&D Systems, USA), rat anti-Endomucin (14-5851-82, Thermo Fisher Scientific, USA), rabbit anti-glutamine synthetase (G2781, Sigma-Aldrich, Germany), goat anti-Arginase I (sc-18351, Santa Cruz, USA), goat anti-RhBg (PA5-19369, Thermo Fisher Scientific, USA), Alexa-Fluor 488, Alexa-Fluor 647 and Cy3-conjugated secondary antibodies were purchased from Dianova (Germany).

### Liver function test

Blood samples were collected in heparinized tubes and allowed to clot at 4 °C. The serum level of alanine transaminase (ALT) was measured using an ELISA kit according to the manufacturer’s instructions (USCN life, USA).

### RNAseq analysis

RNA was prepared using the RNeasy Mini Kit (Qiagen N.V.). Library preparation for bulk 3′-sequencing of poly(A)-RNA was done as described previously^37^. Briefly, barcoded cDNA of each sample was generated with a Maxima RT polymerase (Thermo Fisher) using oligo-dT primer containing barcodes, unique molecular identifiers (UMIs), and an adapter. The 5′ ends of the cDNAs were extended by a template switch oligo (TSO) and full-length cDNA was amplified with primers binding to the TSO-site and the adapter. cDNA was tagmented with the Nextera XT kit (Illumina) and 3′-end-fragments finally amplified using primers with Illumina P5 and P7 overhangs. In comparison to Parekh *et al*. the P5 and P7 sites were exchanged to allow sequencing of the cDNA in read1 and barcodes and UMIs in read2 to achieve a better cluster recognition. The library was sequenced on a NextSeq 500 (Illumina) with 75 cycles for the cDNA in read1 and 16 cycles for the barcodes and UMIs in read2.

Annotations and the reference genome from the Gencode release M15 were derived from the Gencode homepage (https://www.gencodegenes.org/). Dropseq tool v1.12 was used for mapping the raw sequencing data to the reference genome. The resulting UMI filtered count matrix was imported into R v3.4.4 and differential gene expression analysis was conducted with DESeq2^38^. A gene was called differentially expressed if the adjusted p-value was below 0.05 and the absolute log2 fold change was 0.7. Pathway analysis was conducted with EnrichR^39^ within the Reactome database. Pathways with a FDR-level of 0.05 were considered to be statistically significant. Gene level differences for genes being regulated between time points and located in selected pathways are shown as heatmap.

GSEA analysis was performed with a preranked gene list. The rank of a gene was determined according to the following formula:

$$\begin{array}{rcl}{\rm{Rank}}\,(gen{e}_{i}) & = & -lo{g}_{10}\,{\rm{p}}\,{\rm{value}}\,(gen{e}_{i})\times \,lo{g}_{2}\\  &  & \times \,{\rm{Fold}}\,{\rm{Change}}\,(gen{e}_{i})\,{\rm{for}}\,{\rm{all}}\,{\rm{genes}}\,{\rm{i}}.{\rm{n}}.\end{array}$$

Gene Set Variation Analysis was performed with the Bioconductor Package GSVA v1.28.0^40^ after rlog transformation of the UMI filtered Countmatrix within DESeq2. The GSEA Hallmark geneset from the MsigDB database v6.2 was downloaded from http://software.broadinstitute.org/gsea/msigdb/index.jsp and was used as input for GSVA. Resulting Pathway Scores were tested with a ranked based ANOVA for association with the genotype at timepoint 48 hours. P-values were adjusted with the Benjamini Hochberg procedure. We consider a geneset to be positively associated with a genotype at an FDR level of 0.1. Z-score transformed scores for pathways associated with genotype are displayed as heatmap.

### Statistical analysis

Statistical analysis was performed using the GraphPad Prism software (5.0a; Graph- Pad Software, Inc., San Diego, CA). Variation is always indicated using standard error presented as mean ± SEM. Continuous data were tested for normality and analysed by Unpaired Student’s *t*-tests, Mann**-**Whitney *U* test or one-way ANOVA, as appropriate. Statistical significance is displayed as *p < 0.05, **p < 0.01 or ***p < 0.001 unless specified otherwise. In all experiments, no mice were excluded from analysis after the experiment was initiated. Image analysis for the quantification of cell proliferation was blinded.

## Supplementary information

Supplement

## Data Availability

The datasets generated during and/or analysed during the current study are available from the corresponding authors on reasonable request.
