# Supplementary material for: Brg1 promotes liver regeneration after partial hepatectomy via regulation of cell cycle
Source: Sci Rep. 2019 Feb 20;9:2320. doi: 10.1038/s41598-019-38568-w (PMC6382836; doi:10.1038/s41598-019-38568-w)
Supplement: Supplementary file 1 — Supplement [file 41598_2019_38568_MOESM1_ESM.pdf]

Brg1 promotes liver regeneration after partial hepatectomy via regulation of cell cycle

Baocai Wang[1,2], Benedikt Kaufmann[1], Thomas Engleitner[3], Miao Lu[2], Carolin Mogler[4], Victor Olsavszky[5], Rupert Öllinger[3], Suyang Zhong[6], Cyrill Geraud[5], Zhangjun Cheng[2] , Roland R. Rad[3], Roland M. Schmid[6], Helmut Friess[1], Norbert Hüser[1], Daniel Hartmann[1+]*, Guido von Figura[6+]*

1 Department of Surgery, TUM School of Medicine, Klinikum rechts der Isar, Technical University of Munich, Munich, 81675, Germany

2 Department of General Surgery, the Affiliated Zhongda Hospital, School of Medicine, Southeast University, Nanjing, 210000, China

3 Institute of Molecular Oncology and Functional Genomics, Department of Medicine II and TranslaTUM Cancer Center, Klinikum rechts der Isar, Technical University of Munich, Munich, 81675, Germany

4 Institute of Pathology, TUM School of Medicine, Klinikum rechts der Isar, Technical University of Munich, Munich, 81675, Germany

5 Department of Dermatology, Venereology, and Allergology, University Medical Center and Medical Faculty Mannheim, Heidelberg University and Center of Excellence in Dermatology, Mannheim, 68135, Germany

6 Department of Medicine II, TUM School of Medicine, Klinikum rechts der Isar, Technical University of Munich, Munich, 81675, Germany

[+] These authors contributed equally

Figure legends

Figure S1

(A) ALT levels were measured using serum samples of 2-month-old Control and Brg1 KO mice, n=3. (B,C) Liver weight and body weight of 2-month-old Control and Brg1 KO mice, n=6. (D,F) Representative immunofluorescence images for Glutamine synthetase (GS)/Arginase-1 (Arg-1), GS/Lymphatic vessel endothelial hyaluronan receptor 1 (Lyve1)/Endomucin (Emcn) and Rh Family B Glycoprotein (Rhbg) of the liver of 2-month-old Control and Brg1 KO mice, n=3. GS und RhbG show staining pattern in pericentral hepatocytes and Arg1 in the periportal hepatocytes. Liver endothelial cells show a zonated expression pattern as well, with Endomucin (EMCN) in pericentral liver sinusoidal endothelial cells (LSECs) and central vein endothelial cells (CVECs), and LYVE1 in midzonal LSECs[S1].

Figure S2

(A) Representative H&E staining of Control and Brg1 KO mice at 40h after PH. (B) ALT levels were measured using serum samples of post-PH Control and Brg1 KO mice, n>4.

Figure S3

Heatmap of significantly regulated cell cycle pathway related genes analysed in the liver of Control and Brg1 KO mice at 48h after PH. Shown is the z-scaled gene expression for genes, being significantly regulated between time points 0h and 48h , within both the Control and Brg1 KO group, and overlap with the cell cycle pathway annotation from Reactome.

Figure S4

Full length gels of all Western blots shown in the figures.

Table Legends

Table S1

Differences in gene expression levels between time points 4h, 24h, 40h, and 48h post-PH and time point 0hrs for both Control and Brg1 KO mice. For each gene, the baseline gene expression, the log2 fold change and the associated adjusted p-value is reported.

Table S2

Pathway analysis results for differentially expressed genes at time points 4h, 24h, 40h and 48h post-PH compared to baseline expression pre-PH for Control and Brg1 KO mice.

Reference

S1. Leibing, T, et al. Angiocrine Wnt signaling controls liver growth and metabolic maturation in mice. Hepatology; 10.1002/hep.29613(2017).

Figure S1

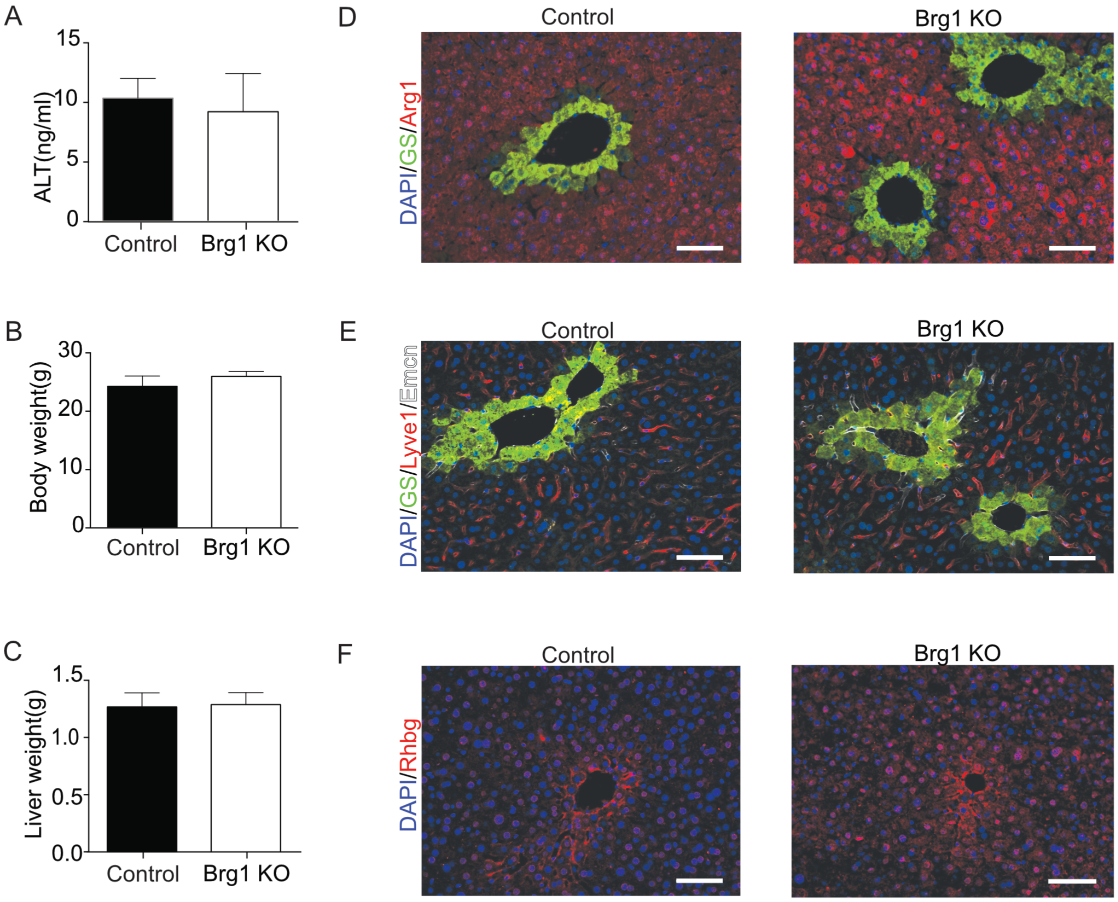
A
ALT(ng/ml)
15
10
5
0
Control
Brg1 KO
B
Body weight(g)
30
20
10
0
Control
Brg1 KO
C
Liver weight(g)
1.5
1.0
0.5
0.0
Control
Brg1 KO
D
Control
Brg1 KO
DAPI/GS/Arg1
E
Control
Brg1 KO
DAPI/GS/Lyve1/Emcn
F
Control
Brg1 KO
DAPI/Rhbg

Figure S2

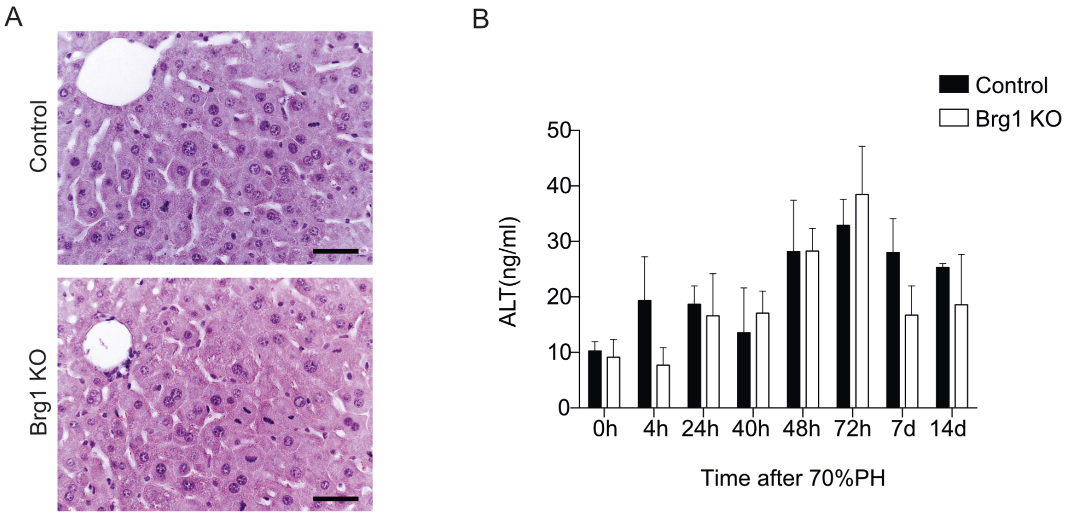
A
Control
Brg1 KO
B
Control
Brg1 KO
ALT(ng/ml)
50
40
30
20
10
0
0h
4h
24h
40h
48h
72h
7d
14d
Time after 70%PH

Figure S3

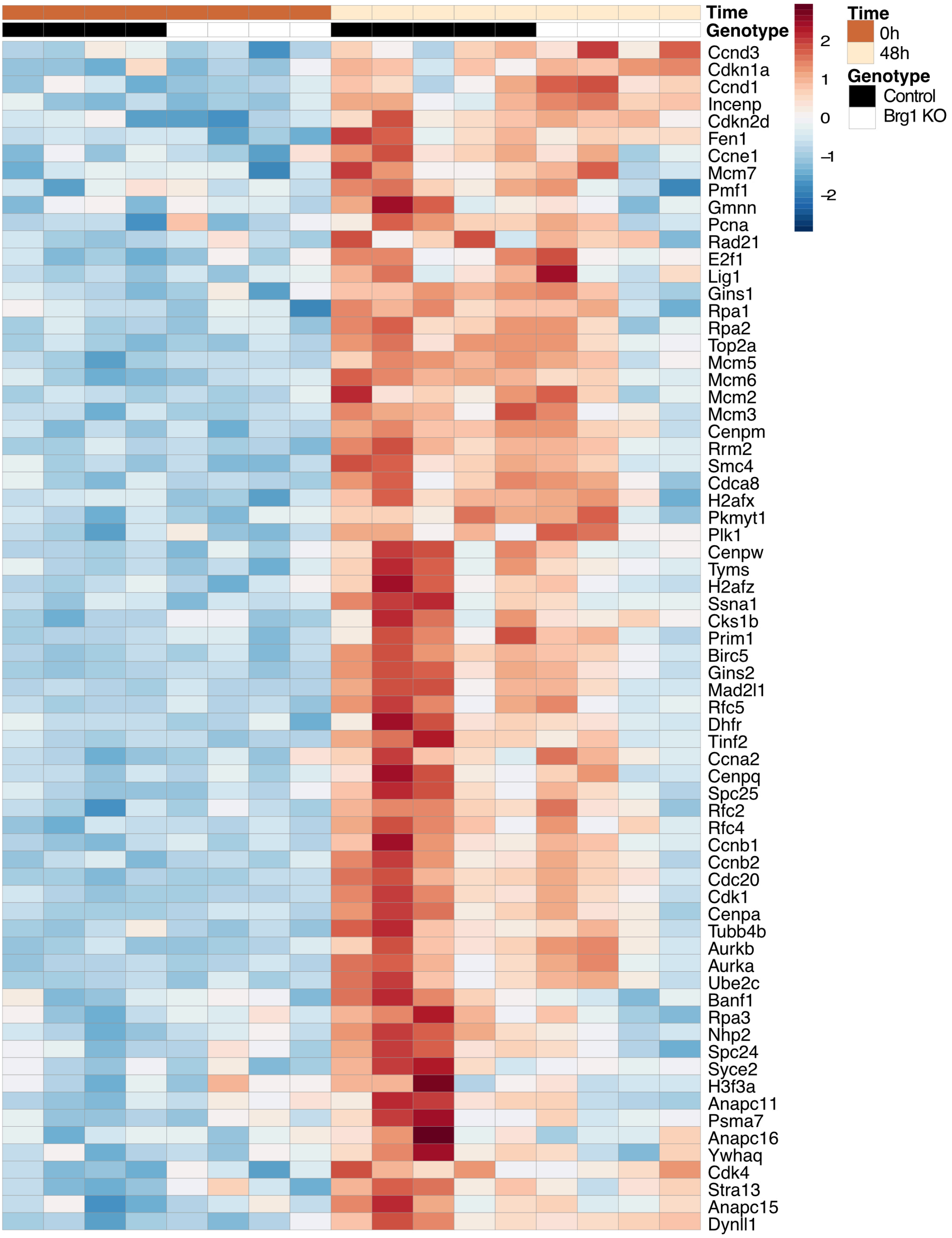
Time
Genotype
Ccnd3
Cdkn1a
Ccnd1
Incenp
Cdkn2d
Fen1
Ccne1
Mcm7
Pmf1
Gmnn
Pcna
Rad21
E2f1
Lig1
Gins1
Rpa1
Rpa2
Top2a
Mcm5
Mcm6
Mcm2
Mcm3
Cenpm
Rrm2
Smc4
Cdca8
H2afx
Pkmyt1
Plk1
Cenpw
Tyms
H2afz
Ssna1
Cks1b
Prim1
Birc5
Gins2
Mad2l1
Rfc5
Dhfr
Tinf2
Ccna2
Cenpq
Spc25
Rfc2
Rfc4
Ccnb1
Ccnb2
Cdc20
Cdk1
Cenpa
Tubb4b
Aurkb
Aurka
Ube2c
Banf1
Rpa3
Nhp2
Spc24
Syce2
H3f3a
Anapc11
Psma7
Anapc16
Ywhaq
Cdk4
Stra13
Anapc15
Dynll1
2
1
0
-1
-2
Time
0h
48h
Genotype
Control
Brg1 KO

Figure S4

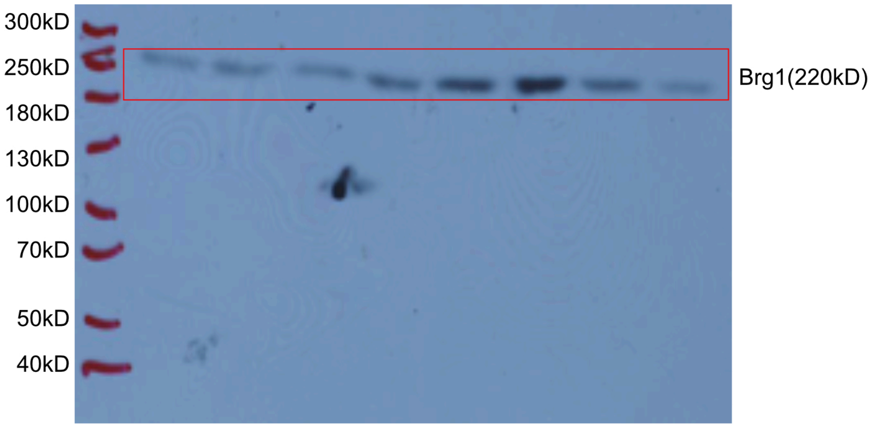

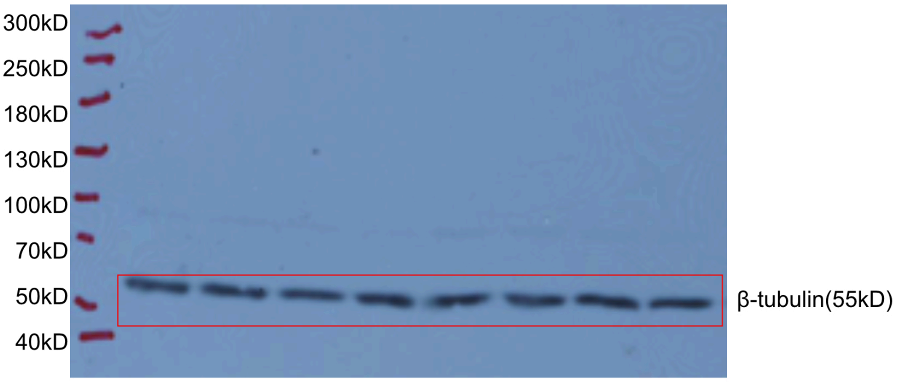

Areas of the gel shown in Figure 1A are marked in red.

The membrane was probed first with anti-Brg1 and subsequently with anti-β-tubulin antibodies. Predicted molecular weights for Brg1 and β-tubulin are indicated.

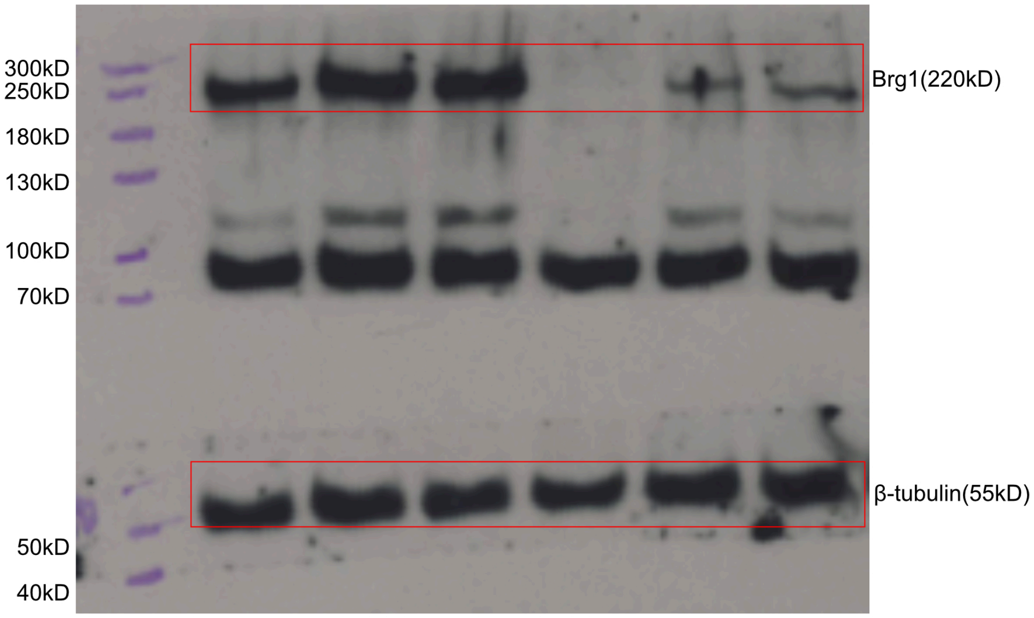

Areas of the gel shown in Figure 2A are marked in red.

The membrane was probed with anti-Brg1 and anti-β-tubulin antibodies. Predicted molecular weights for Brg1 and β-tubulin are indicated.

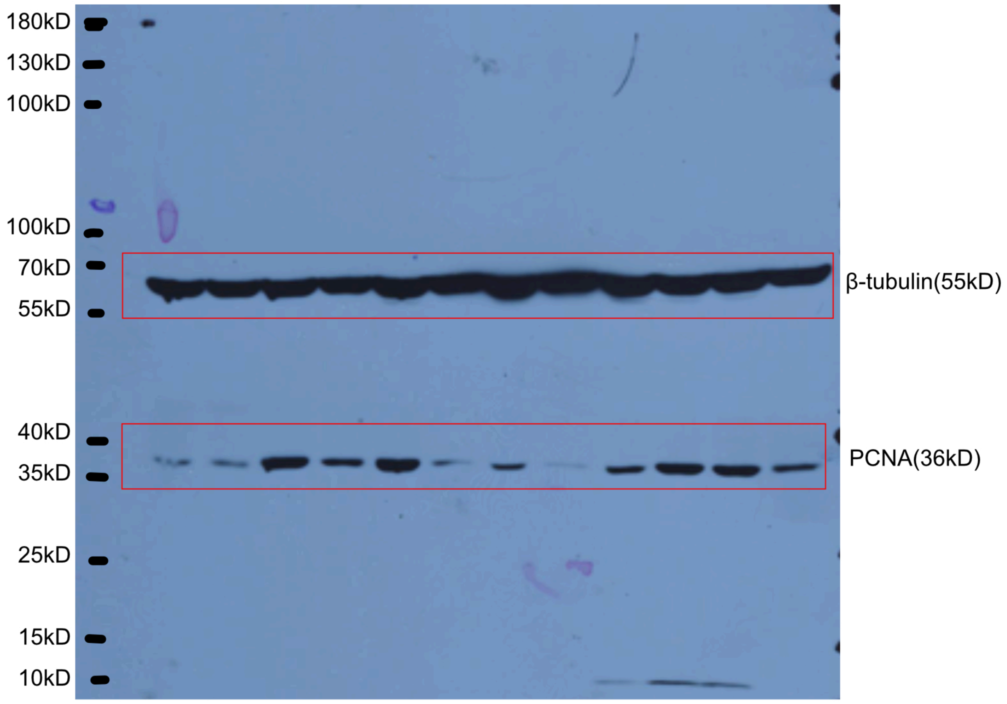

Areas of the gel shown in Figure 4A part1 are marked in red.

The membrane was probed with anti-PCNA and anti-β-tubulin antibodies. Predicted molecular weights for PCNA and β-tubulin are indicated.

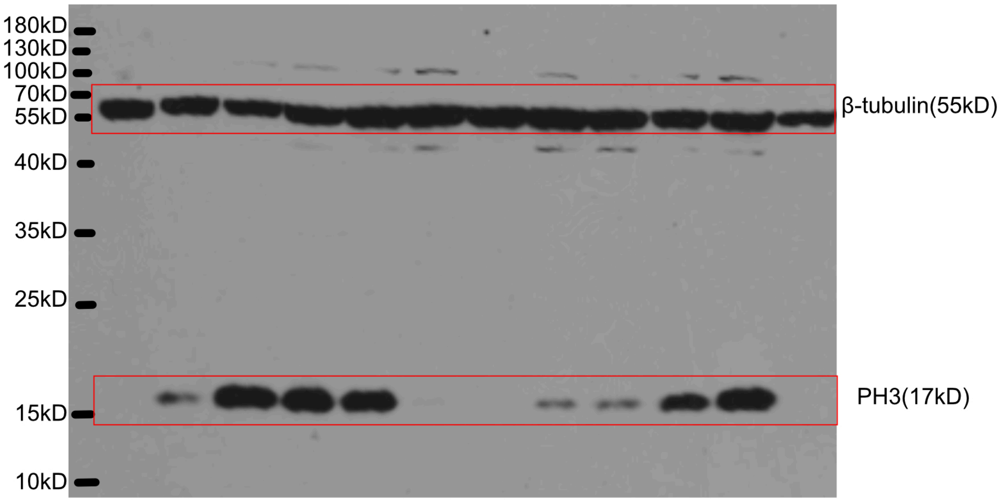

Areas of the gel shown in Figure 4A part2 are marked in red.

The membrane was probed with anti-β-tubulin and anti-PH3 antibodies.

Predicted molecular weights for PH3 and β-tubulin are indicated.

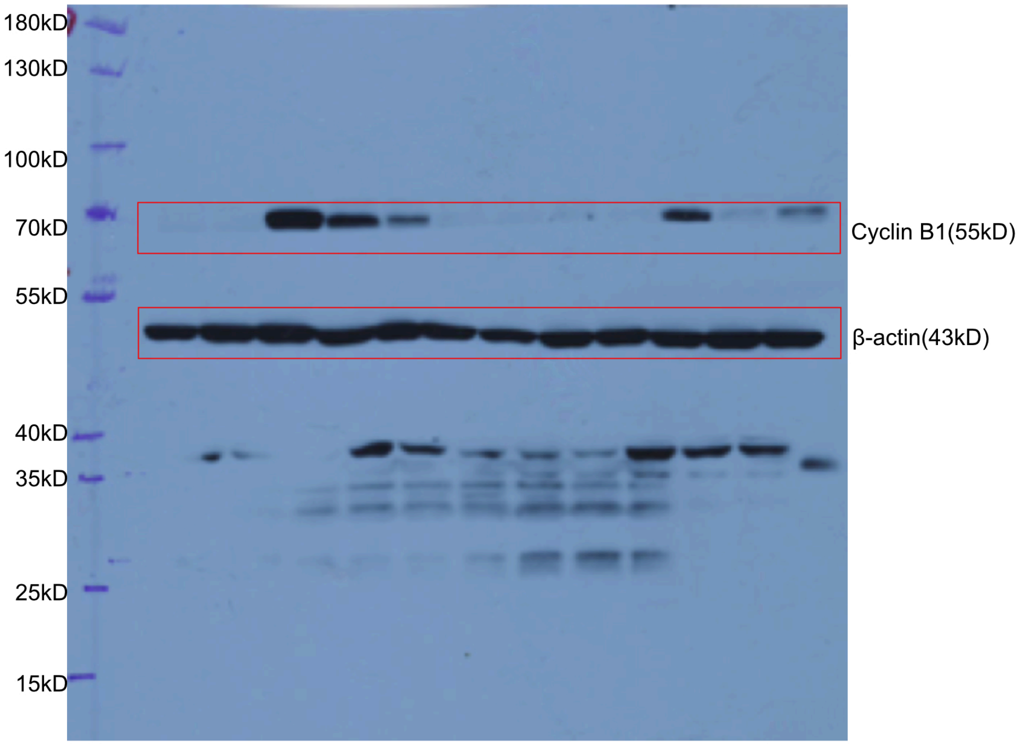

Areas of the gel shown in Figure 6B part1 are marked in red.

The membrane was probed with anti-Cyclin B1 and anti-β-actin antibodies. Predicted molecular weights for Cyclin B1 and β-actin are indicated.

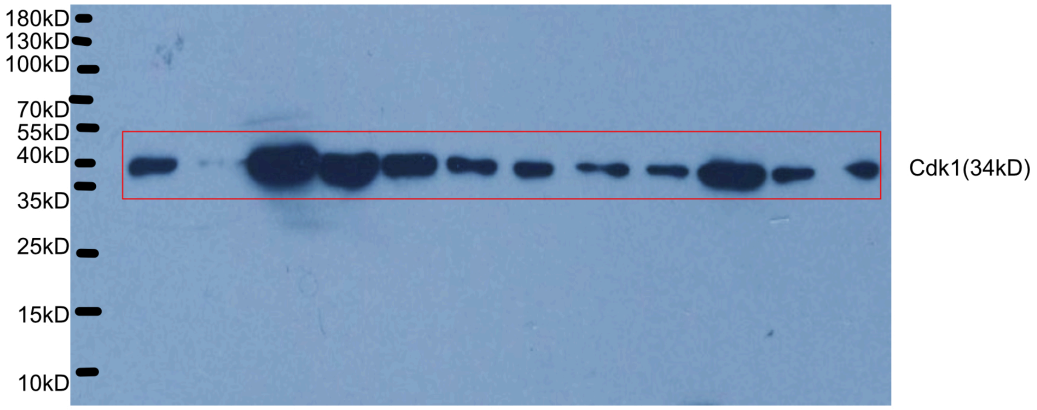

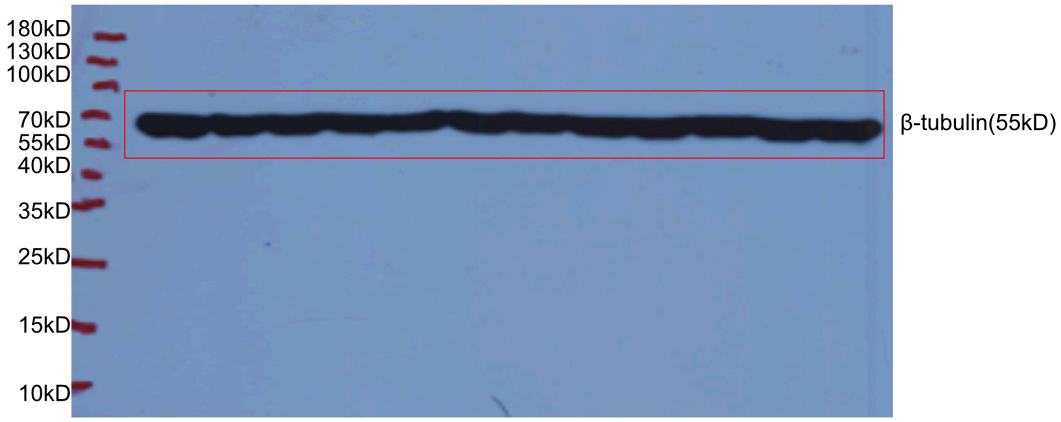

Areas of the gel shown in Figure 6B part2 are marked in red.

The membrane was probed first with anti-Cdk1 and subsequently with anti-β-tubulin antibodies. Predicted molecular weights for Cdk1 and β-tubulin are indicated.

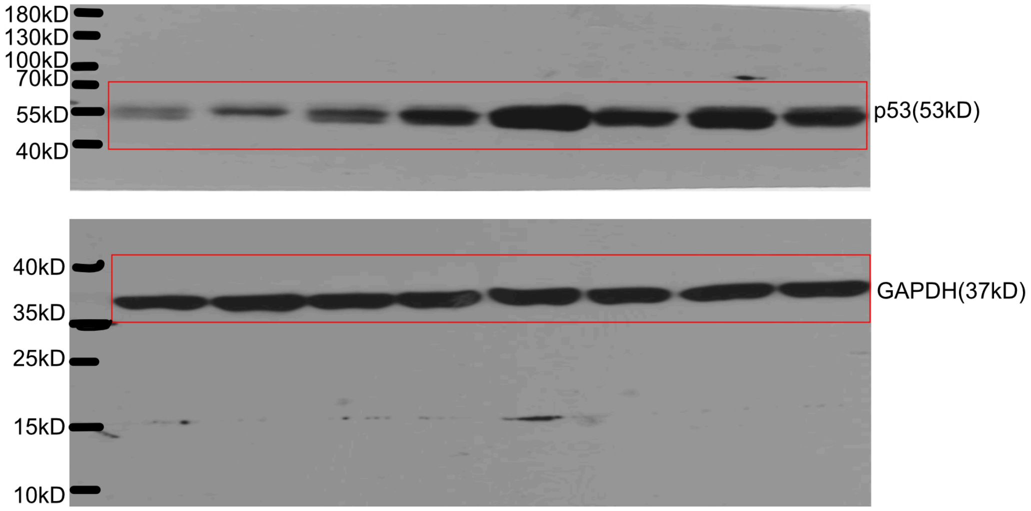

Areas of the gel shown in Figure 7B are marked in red.

The membrane was probed with anti-p53 and anti-GAPDH antibodies. Predicted molecular weights for p53 and GAPDH are indicated.
